# The social and economic burden of stroke survivors in Italy: a prospective, incidence-based, multi-centre cost of illness study

**DOI:** 10.1186/1471-2377-12-137

**Published:** 2012-11-14

**Authors:** Giovanni Fattore, Aleksandra Torbica, Alessandra Susi, Aguzzi Giovanni, Giancarlo Benelli, Marianna Gozzo, Vito Toso

**Affiliations:** 1 Centre for Research on Health and Social Care Management (CERGAS) and SDA Bocconi, Università Bocconi, Milan, Italy; 2 Local Health Unit, Modena, Italy; 3 AstraZeneca S.p.A, Basiglio, Italy; 4 Ospedale San Bortolo, Vicenza, Italy

**Keywords:** Cost of illness, Costs and cost analysis, Stroke, Italy

## Abstract

**Background:**

The aim of this study was to estimate the one-year societal costs due to a stroke event in Italy and to investigate variables associated with costs in different phases following hospital admission.

**Methods:**

The patients were enrolled in 44 hospitals across the country and data on socio-demographic, clinical variables and resource consumption were prospectively surveyed for 411 stroke survivors at admission, discharge and 3, 6 and 12 months post the event. We adopted a micro-costing procedure to identify cost generating components and the attribution of appropriate unit costs for three cost categories: direct healthcare, direct non-healthcare (including informal care costs) and productivity losses. The relation between costs of stroke management and socio-demographic and clinical characteristics as well as disability levels was evaluated in a series of bivariate analyses using non parametric tests (Mann Whitney and Kruskal-Wallis). Multiple linear regression analyses were performed to determine predictors of costs incurred by stroke patients during the acute phase and follow-up of 1 year.

**Results:**

On average, one-year healthcare and societal costs amounted to €11,747 and € 19,953 per stroke survivor, respectively. The major cost component of societal costs was informal care accounting for € 6,656 (33.4% of total), followed by the initial hospitalisation, (€ 5,573; 27.9% of total), rehabilitation during follow up (€ 4,112; 20.6 %), readmissions (€ 439) and specialist and general practioner visits (€ 326). Mean drug costs per patient over the follow-up period was about € 50 per month. Costs associated to the provision of paid and informal care followed different pattern and were persistent over time (ranging from € 639 to € 597 per month in the first and the second part of the year, respectively). Clinical variables (presence of diabetes mellitus and hemorrhagic stroke) were significant predictors of total healthcare costs while functional outcomes (Barthel Index and Modified Ranking Scale scores) were significantly associated with both healthcare and societal costs at one year.

**Conclusions:**

The significant role of informal care in stroke management and different distribution of costs over time suggest that appropriate planning should look at both incident and prevalent stroke cases to forecast health infrastructure needs and more importantly, to assure that stroke patients have adequate “social” support.

## Background

Stroke is currently the second leading cause of death in the Western world, ranked after heart disease and before cancer [1]. The annual incidence of stroke in Italy is approximately 130,000 individuals with primary stroke and an additional 50,000 individuals who suffer from recurrent stroke [2]. Being a disease with long-term consequences, stroke creates considerable social and economic burden to individuals and society. Several costs of stroke studies have been carried out in the last decade with the aim of estimating this burden in different countries [3-9]. Comparisons of the estimates obtained across countries present notable differences, mainly because of differences in data collection, population studies, period of observation and often definition of cost components [10]. Nevertheless, some common findings emerged from the recent review on the international evidence regarding the composition of costs and their relative importance within different cost categories and most importantly cost drivers [11,12]. In a nutshell, the majority of available studies agree high consumption of health care resources is mainly due to initial hospitalization and rehabilitation services. The acute phase generally accounts for more than 50% of the total costs over the first year post the event (with acute hospitalization costs ranging from 30% to 40%); the remaining costs mostly concern rehabilitation services (ranging between 15% and 35%), thus suggesting the importance of follow-ups within health policy maker’ agendas [3,5,9,11-15]. As to drivers of costs, a common result is that stroke severity is a cost predictor in all phases of care and neurologist/specialist wards cost more than general medicine [14,16,17].

To offer guidance to policy makers, technological advancements and patients’ care changes over time need to be understood. Consequently, the production of up to date relevant evidence on the use of resources for stroke is essential to provide guidance for health planning and the allocation of financial resources. In addition to providing valuable insights into social and economic burden of stroke in different countries, this study allows of a generalization of findings at a national level whereas the majority of available cost studies in Europe are limited to single or only a few hospitals. Similarly, several studies show the average costs of patients with stroke, but do not provide precise estimates of the stroke-specific costs [12]. A further limitation of the current literature is that it mainly focuses on healthcare costs associated with stroke while neglecting the costs related to informal care or productivity losses, the two cost categories that represent the most significant burden on lifetime costs of stroke [12].

This study overcomes these limits by conducting a large scale, multicentre, prospective cost of illness analysis in Italy, with the primary aim of estimating stroke health and societal costs in the period 2005–2007. The Italian context is of particular interest due to its decentralized health system which allows wide autonomy to regions in adopting different models in organization and provision of care to stroke patients. This regional differentiation takes place within the framework of the Italian National Health Service (NHS) which provides to all citizens full coverage of hospital and outpatient services and financial support to medical costs delivered in nursing homes [18]. The secondary objective of the study is to identify the main clinical and demographic predictors of costs of survivors at one year post stroke event.

## Methods

### Study population

In order to reach the aforementioned objectives, we designed an observational, prospective, incidence-based, multi-centre cost of illness study. The Italian territory was divided in 3 macro-areas and in the study protocol we imposed that the ratio between the resident population and the number of patients to enrol was the same across all areas. For each area we identified hospitals by inclusion of at least one medicine, one neurology and one stroke unit ward to reflect different practices of care in Italy [5]. Patients inclusion criteria for enrolment were defined with the clinician participation and included the following: diagnosis of stroke event (primary and recurrent) supported by Computerized Tomography (CT) scan and/or Magnetic Resonance (MRI) for patients above 18 years of age (International classification of disease −9 [ICD-9] codes 430, 431, 433, 434 and 436). Informed Consent was required to include the patient in the study.

In each centre patients were selected consecutively to be screened for inclusion criteria. Patients were excluded from the study if evidence of any of the following conditions occurred throughout hospitalisation: 1) presence of subarachnoid haemorrhage; 2) severe pathologies with unfavourable 1-year prognosis (cancers, fatal renal, hepatic or respiratory insufficiency); 3) disabling and progressive neurological diseases (multiple sclerosis, Parkinson’s disease); 4) dementia (diagnosed according to diagnostic and statistical manual of mental disorders [DSM-IV] criteria) and 5) refusal or withdrawal of patient informed consent to participate in the study. According to the protocol all inclusion and exclusion criteria had to be checked by a neurologist.

Patients enrolment lasted 3-month in all 44 hospitals distributed across the country (21 North, 9 Center, and 14 South [Table 1]). After the enrolment period, patients were followed up for one year. The study was approved by the local ethics committees of all 44 participating hospitals and was conducted from August 2005 to March 2007.

**Table 1:** Sample geographic distribution

**Macro-area**	**Regions**	**Number of patients enrolled in the study**	**Population (000)**	**Ratio**
North	V.d’Aosta, Piemonte, Liguria, Lombardia, P.A Bolzano, P.A Trento, Veneto, Friuli V.G., Emilia-Romagna	252	27,763	0.0091
Centre	Toscana, Marche, Umbria Abruzzo, Molise, Lazio	101	11,950	0.0085
South	Campania, Puglia, Basilicata Calabria, Sicilia, Sardegna	193	20,912	0.0092
Italy		546	60,626	0.0091

### Study tools

Five questionnaires were designed to collect socio-demographic (age, gender, place of residence, employment and living status), clinical and economic data on patients and caregivers. These questionnaires were administered at hospital admission, at discharge, and 3, 6 and 12 months after the event. All questionnaires were filled in by hospital staff during patient admissions and recurrent visits at the hospital centres. We undertook two investigators’ meetings to present the protocol of the study and to review all the study tools.

Clinical variables included co-morbidities and type of stroke (ischemic or hemorrhagic; primary or recurrent). The Bamford Scale (BS) classification was used during hospitalisation to categorise patients in lacunar stroke syndrome (LACS), partial anterior circulation stroke (PACS), posterior circulation stroke (POCS) and total anterior circulation stroke (TACS). Four different scales were used to assess clinical outcomes. Stroke severity was assessed at admission using the National Institute for Health Stroke Scale (NIHSS). Barthel Index (BI) and Modified Ranking Scale (mRS) were used to measure physical disability of stroke patients at admission, discharge, and 3, 6 and 12 months post the event.

### Cost analysis

Cost data were collected during initial hospitalisation and throughout one year follow-up. We adopted a micro-costing procedure to identify cost generating components and to attribute appropriate unit costs for three cost categories: direct healthcare, direct non-healthcare (including informal care costs) and productivity losses. All questionnaires were designed to specifically capture only the stroke-specific costs. In line with the social perspective of the analysis, both direct and productivity costs for the year following the stroke event were estimated. Direct healthcare costs included the hospitalization in the acute phase, additional stroke-related hospital admissions, clinical consultations, diagnostic tests and procedures, domiciliary care, medical therapies, rehabilitation services and other healthcare costs (i.e. appliances, aids). Direct non-healthcare costs included transportation costs and out-of-pocket expenses for paid social care. In addition, we estimated the costs of informal care provided by family members, friends and other voluntary personnel. To fully evaluate the economic loss due to stroke we estimated productivity costs as the value of forgone time that would have otherwise been spent for productive activities if the patient did not suffered from the stroke event.

The direct cost of index hospitalization was assessed by multiplying number of units of resources used while in hospital (eg. drugs, imaging and laboratory exams, consumables, medical procedures, consultation visits, inpatient rehabilitation) and unit costs provided by hospital accounting departments when available. For estimating hospital personnel and overhead costs Italian data collected in a major European study estimating costs according to a pre-determined protocol were used [4].

Information concerning the use of resources in natural terms during follow-up (e.g. number of general practitioner [GP] visits, days of work lost, number of hours of informal care) were collected through the administration of questionnaires. Unit costs were mainly estimated through regional and national tariffs used to fund providers by the Italian NHS. Transport costs were estimated directly from the patients (e.g. the cost of public transportation) or on the basis of an estimated cost per kilometre travelled.

Cost per hour of informal care was estimated according to the replacement cost approach, the hourly gross cost of social care as set by the National Labour Contract in 2007. Productivity costs were estimated using the human capital approach. Each lost working day was valued equivalent to the mean daily income associated to the main categories of workers identified by national statistics.

### Statistical analysis

All statistical analyses were performed using the software package Data Analysis and Statistical Software [STATA] 11.0 (StataCorp, College Station, TX, USA). Statistical tests used to compare baseline characteristics of survivors to patients lost to follow up were Student t test for continuous and chi-square for categorical variables. In order to evaluate costs of stroke management in relation to socio-demographic and clinical characteristics as well as disability levels, we performed a series of bivariate analyses using non parametric tests (Mann Whitney and Kruskal-Wallis). Multiple linear regression analyses were performed to determine predictors of costs incurred by stroke patients during the acute phase (model 1) and follow up (model 2 for healthcare costs and model 3 for societal costs). In each model we used a set of predictors capturing socio-demographic conditions (age, gender, living condition), geography (north, centre and south), major clinical events, risk factors, organizational settings (stroke units, neurology ward and medicine ward), functional outcomes according to NIHSS (at admission) and BI categories, and disability (mRS). The values obtained from the scales referred to the beginning of the reference period (for example in multiple regression model 2 on healthcare costs during follow up values referred to BI and mRS obtained at discharge). We kept all the possible predictors in the three models regardless the significance of the coefficients. Due to non-normal distribution of cost data all costs were logarithmically transformed to run the three regression models [16]. This transformation allowed to use parametric methods and resulted in models with better goodness of fit.

## Results

We enrolled and collected data at hospital discharge for 546 patients. General socio-demographic characteristics remained basically unchanged for the subsample of 411 patients who survived after 12 months and were not lost at follow-up (Table 2). At enrolment, 84% of patients had a cerebral infarction diagnosis, 43% were females and 69% were over 65 years of age. The recruitment of patients followed planned geographical macro-area quotas as the patients/population ratio varied less than 10% around the national mean. Death was registered for 44 patients (8.1%) which were significantly older (80 vs 69 years, p<0.001), retired (84 vs 68% , p=0.010) and more likely to be female (61 vs 41%, p=0.008). For 91 individuals we could not collect complete data because the patients either were lost to follow-up or withdrew from the study. Their baseline characteristics were not significantly different from the surveyed sample (Table 2).

**Table 2:** Socio-demographic sample characteristics

	**At enrolment**		**At 12 months follow up**		**Lost to follow up**		P value*	**Deaths**		P value**
	**n= 546**		**N=411**		**n=91**			**n=44**		
	**N./ mean**	**%/SD**	**N./ mean**	**%/SD**	**N./ mean**	**%/SD**		**N./ mean**	**%/SD**	
Age mean (SD)	69.5	12.9	69	12.9	68.4	12.1	0.919	80	8.9	<0.001
Median (IQR)	72.0	16	71.0	16	78.0	17	0.824	88	13.5	<0.001¥
Location							0.500			0.382
North	252	46.2%	195	47.4%	41	45.1%		16	36.4%	
Centre	101	18.5%	78	19.0%	14	15.4%		9	20.5%	
South and islands	193	35.3%	138	33.6%	36	39.6%		19	43.2%	
Gender							0.970			0.008
Female	232	43%	168	41%	37	40.7%		27	61%	
Employment status							0.581			0.010
Retired	372	68.1%	278	67.6%	57	62.6%		37	84.1%	
Employed	106	19.4%	83	20.2%	22	24.2%		1	2.3%	
Other	68	12.5%	50	12.2%	12	13.2%		6	13.6%	
Living status							0.970			0.114
Lives with family	446	81.7%	339	82.5%	76	83.5%		31	70.5%	
Lives by her/himself	80	14.7%	57	13.9%	12	13.2%		11	25.0%	
Other	20	3.7%	15	3.6%	3	3.3%		2	4.5%	

*comparison patients with full follow up (n=411) to lost to follow up (n=91).

** comparison patients with full follow up (n=411) to dead (n=44).

¥ Mann Whitney test.

At enrolment, 464 patients (84%) were diagnosed with an ischemic stroke. Compared to survivors patients who died during the follow-up had higher prevalence of all cardiovascular comorbidities (hypertension, atrial fibrillation and diabetes mellitus), as well as previous strokes (34 vs. 10%, p<0.001) and were more likely to have TACS (46 vs. 18% using BS**).** On the contrary, 91 patients lost to follow up had clinical characteristics at discharge similar to the 411 patients included in the subsequent economic analysis (Table 3).

**Table 3:** Clinical sample characteristics

	**At enrolment**		**At follow up 12 months**		**Lost to follow up**		P value*	**Dead**		P value**
	**n= 546**		**n=411**		**n=91**			**n=44**		
	**N./mean**	**%/SD**	**N./mean**	**%/SD**	**N./ mean**	**%/SD**		**N./mean**	**%/SD**	
Hypertension	383	70.1%	277	67.4%	71	78.0%	0.047	35	79.5%	0.155
Atrial Fibrillation	104	19.0%	72	17.5%	15	16.5%	0.814	17	38.6%	**0.001**
Diabetes Mellitus	118	21.6%	88	21.4%	18	19.8%	0.731	12	27.3%	0.341
Previous strokes	62	11.4%	39	10%	8	8.8%	0.836	15	34.1%	<0.001
Previous TIA	54	9.9%	40	9.7%	4	4.4%	0.103	10	22.7%	0.003
Previous AMI	54	9.9%	38	9.2%	9	9.9%	0.849	7	15.9%	0.163
Ischemic	464	84%	352	85.6%	76	83.5%	0.604	36	81.8%	0.540
Bamford Classification §							0.295			<0.001
PACS	177	32.4%	126	30.7%	37	40.7%		14	31.8%	
LACS	181	33.2%	146	35.5%	29	31.9%		6	13.6%	
POCS	82	15.0%	65	15.8%	13	14.3%		4	9.1%	
TACS	106	19.4%	74	18.0%	12	13.2%		20	45.5%	
NIHSS – at admission Mean (SD)	7.2	6.5	6.7	6.0	6.2	5.7	0.403	13.2	9.6	<0.001
Median (IQR)	5.0	7.0	5.0	6.0	4.0	8.0	0.157¥	13.0	14.0	<0.001¥
Barthel Index mean-admission	52.3	36.4	54.3	35.0	58.1	38.1	0.363	19.4	30.1	<0.001
Barthel Index mean-discharge	67.5	36.5	70.9	33.1	68.7	35.8	0.566	26.2	37.4	<0.001
Barthel Index category at discharge							0.588			**<0.001**
Indipendence (96–100)	189	34.6%	215	52.3%	37	40.7%		4	9.1%	
Mild dependence (75–95)	106	19.4%	77	18.7%	17	18.7%		3	6.8%	
Moderate dependence (46–74)	84	15.4%	58	14.1%	12	13.2%		2	4.5%	
Severe dependence (0–45)	145	26.6%	56	13.6%	24	26.4%		27	61.4%	
N.A.	22	4.0%	5	1.2%	1	1.1%		8	18.2%	
Modified Ranking Scale at discharge							0.217			**<0.001**
0	50	9.2%	89	21.7%	8	8.8%		4	9.1%	
1	133	24.4%	128	31.1%	28	30.8%		2	4.5%	
2	76	13.9%	41	10.0%	13	14.3%		3	6.8%	
3	94	17.2%	71	17.3%	18	19.8%		1	2.3%	
4	131	24.0%	64	15.6%	11	12.1%		11	25.0%	
5	62	11.4%	18	4.4%	13	14.3%		23	52.3%	

(§) PACS = Lacunar stroke, PACS = Partial anterior circulation stroke, TACS = Total anterior circulation stroke, POCS = Posterior circulation stroke.

*comparison patients with full follow up (n=411) to lost to follow up (n=91).

** comparison patients with full follow up (n=411) to dead (n=44); ¥ Mann Whitney test.

On average, survivors spent 13.5 days in hospital for the stroke event and the length of stay was not significantly different across different settings of care (Table 4). After discharge most of the survivors used GP services (87%), specialist visits (77%) and diagnostic procedures (67%). Total direct healthcare costs per patient amounted to €11,747 (median €6,727, Interquartile Range [IQR] €9,483). About 47% of such costs were due to the first index hospitalisation and rehabilitation services, costing on average €4,112, accounted for another 35%. Non healthcare costs amounted to an average of €8,206 per patient illustrating great variability (median 4,320, IQR €13,425) and are mainly concerned with the economic value of informal care (€ 6,656). Costs of paid care and those attributable to production losses were estimated at €758 and €792, respectively.

**Table 4:** Resources and costs during the first 12 months after the stroke event (survivors)

	**Users**		**Resources per patient**		**Cost per patient (€)**			
	**N.**	**%**	**Mean**	**Sd**	**Mean**	**Sd**	**Median**	**IQR**
**Healthcare costs**								
N. of days of the index hospitalization (mean)	411	100%	13.5	11.9	5,573	4,515	4,700	27,65
Stroke Unit	124	30%	12.2	7.2	5,464	2,702	4,713	2,914
Neurology	189	46%	14.2	10.2	6,241	6,028	5,013	3,195
General Medicine	98	24%	10.6	6.2	4,423	2,059	4,127	2,736
Post-acute phase								
Inpatient *days*	43	11%	1.1	4.1	439	1,693	0	0
Rehabilitation sessions	198	48%	29.5	51.1	335	580	34	409
Inpatient rehabilitation (days)	96	23%	12.7	28.8	3,777	8534	0	0
Imaging	274	67%	2.7	3.7	144	250	63	193
GPs visits	364	89%	8.9	9.2	146	153	116	149
Specialists visits	317	77%	7.0	18.0	180	425	33	116
Drugs	391	95%			628	1377	272	425
Nursing home	33	8.0%	7,7	38.1	336.0	1663.2	0	0
Other costs					524.2	1672.3	0	0
**Total healthcare costs**					11,747	11,250	6,727	9,483
Non-healthcare costs								
Informal care (hours per day)	183	45%	7.8	5.2	6,656	11,051	1800	
Paid care (hours per day)	60	15%	1.2	0.1	758	2,232	0	0
Production losses	25	6.1%			792	28	0	78
**Total societal costs**					19,953	18,114	13,714	22,058

Total follow up costs decreased over time from €2,157 per month in the first three months and post the event to €**762** per month at the end of the observation period (Table 5). The composition of costs varied greatly over time. In the first period healthcare costs were 63.8 % of the total while in the last six months they were only 21.2%. Moreover, in the first half of the year following the stroke event inpatient rehabilitation services were the major component of healthcare costs (78.2%) while in the proceeding 6 months of follow up the most important category of healthcare costs were drugs (27.3%). Non-healthcare costs were stable over the period (€639 per month in the first half of the year and € 597 in the second). Contrary to above, production losses in the first 3 months were €439 per patient and dropped to €20 in the second part of the year post stroke.

**Table 5:** Follow up costs per patient across study phases (n= 411, in €)

	**Discharge - 3months**		**3 -6 months**		**6-12 months**	
	**Mean**	**SD**	**Mean**	**SD**	**Mean**	**SD**
Healthcare costs						
Inpatient	106.5	828.4	129.4	721.9	203.4	1078.6
Rehabilitation sessions	130.3	210.7	86.1	196.7	118.7	326.8
Inpatient rehabilitation	3230.8	6848.5	422.3	2663.1	123.8	1406.1
Imaging	65.2	138.8	36.4	102.8	42.3	91.3
GPs visits	42.8	50.6	47.5	71.1	56.1	75.4
Specialists visits	77.7	217.2	46.9	149.5	55.8	253.8
Drugs	178.1	545.5	185.9	664.7	263.7	662.4
Nursing home	139.8	637.8	108.0	635.6	88.2	795.4
Other costs	160.9		8.5		18.1	
*Total healthcare costs*	*4132.0*	*7067.0*	*1071.0*	*3214*	*970.0*	*2182*
Non-healthcare costs						
Informal care	1771.3	2373.2	1719.6	3364.2	3164.85	6485.40
Paid care	128.5	457.4	213.6	672.7	416.1	1252.4
Production losses	439	1983.7	99.17	979.50	20	194.1
Total Follow Up Costs	6470.8	8227.0	3103.34	5054.2	4571.2	7390.2

The results of bivariate analyses displayed in Table 6 give some insight into the association between clinical characteristics and total healthcare costs. Presence of diabetes mellitus was associated with higher healthcare costs but only in the follow up period. Atrial fibrillation appeared positively associated to higher hospitalisation costs (€ 6,700 vs. € 5,334) although this difference was not statistically significant. Significant differences are observed for type of stroke but only in the follow up period. More specifically, while the magnitude of costs during hospitalisation are similar for haemorrhagic and ischemic cases, patients with haemorrhagic stroke have follow-up healthcare costs 2.3 times higher than the ischemic cases leading to significantly higher total healthcare costs (€ 17,383 vs 10,802; p<0.001). Both BI and mRS are strongly associated to healthcare costs over the entire observation period. In the first three months after discharge patients with severe dependency (BI under 45) had healthcare costs almost 9 times higher than those with no dependency (BI over 96). In the last six months of observation such difference was still very evident with the ratio of costs between severe dependency and no dependency close to 4. Healthcare costs were also much higher for patients with severe disability (mRS scale 4–5) in comparison to those with no or mild disability (mRS 0–2): 3,062 vs. € 513 per month in the first three months after discharge and € 364 vs. €86 per month in the last 6 months of the observation period.

**Table 6:** Total healthcare costs by subgroups of survivors across study phases (n=411, cost in €)

	**Index hospitalization**		**Discharge – 3months**		**3 -6 months**		**6-12 months**		**TOTAL**	
	**Mean**	**Median**	**Mean**	**Median**	**Mean**	**Median**	**Mean**	**Median**	**Mean**	**Median**
**Gender**										
Male	5297.3	4803.8	3795.9	367.4	971.7	200.2	884.0	294.5	10948.9	7159.5
Female	5972.3	4689.7	4618.2	457.0	1214.5	250.7	1095.9	313.2	12901.0	6626.0
*p value*		*0.397*		*0.613*		*0.318*		*0.937*		*0.422*
**Diabetes**										
Yes	5624.5	4981.1	4201.6	688.3	1176.2	337.2	1369.3	414.6	12371.7	8253.4
No	5559.2	4675.5	4113.0	357.4	1042.3	201.8	862.0	277.0	11576.6	6546.4
*p value*		*0.701*		*0.025*		*<0.001*		*<0.001*		*0.114*
**Atrial Fibrillation**										
Yes	6700.4	5048.6	3863.9	458.2	1247.3	248.0	1241.9	332.5	13053.5	7684.5
No	5334	4663.8	4188.9	418.1	1033.5	225.9	913.0	296.0	11469.3	6546.4
*p value*		*0.098*		*0.817*		*0.583*		*0.453*		*0.214*
**Previous strokes**										
Yes	5617.8	5180.6	3827.2	712.0	829.1	423.1	1116.7	394.9	11390.8	9172.7
No	5568.5	4668.8	4164.0	416.3	1096.3	223.5	955.3	296.3	11784.2	6666.9
*p value*		*0.212*		*0.295*		*0.035*		*0.364*		*0.519*
**Type of stroke**										
Heamorrhagic	5817.9	5180.6	8021.8	1645.6	2225.4	357.4	1318.1	377.2	17383	12625.1
Ischemic	5532.2	4668.8	3480.0	394.0	877.5	222.0	912.4	294.5	10802	6455.4
*p value*		*0.182*		*<0.001*		*0.001*		*0.224*		*<0.001*
Barthel Index *										
Indipendence (96–100)	4581.9	3694.0	1103.2	207.9	335.8	141.0	502.9	217.1	6240.4	4480.4
Mild dependence (75–95)	4446.2	4053.1	1897.0	365.2	514.4	231.1	798.3	429.7	8194.1	5438.1
Moderate dependence (46–74)	4937.8	4484.3	5488.7	999.1	1788.8	340.6	1807.9	564.7	14823.8	6298.8
Severe dependence (0–45)	6609.4	5563.9	9826.6	8625.5	2733.3	743.1	2083.7	735.7	21552.7	11875.9
*p value*		*<0.001*		*<0.001*		*<0.001*		*<0.001*		*<0.001*
Modified Ranking Scale*										
no or mild disability (0–2)			1225	219.1	342.7	144.4	512.6	223.3	1964.8	683.9
moderate disability (3)			3369.5	507.6	1687.3	457.5	939.2	439.7	5239.1	1738.6
severe diasability (4–5)			9185	8088.3	2334.9	514.1	2181.7	701.3	13381.5	10858.8
*p value*				*<0.001*		*<0.001*		*<0.001*		*<0.001*

*scales at the beginning of the reference period.

Cost predictions were further investigated in the multivariate analysis (Table 7). Only clinical and organizational characteristics predict differences in costs for the acute phase: patients with higher NIHSS and with higher level of dependence according to the BS index have higher costs. Patients admitted to neurology wards or stroke units are more costly than those admitted to general medicine wards. Healthcare costs for the one year follow-up are predicted by demographic, organizational and clinical variables. Patients who were admitted to neurology wards in the acute phase are less costly than those admitted to general medicines and stroke units. Male patients, with diabetes and haemorragic stroke present higher costs. The mRS is strongly associated with costs, whereby moderate and severe patients had higher costs than mild patients by 75% and 121%, respectively.

**Table 7:** Multivariate analyses: drivers of cost in the acute phase (index hospitalisation), healthcare costs and societal costs in the 1-year follow-up after the stroke event

**Dependent variable Log (costs)**	**Model 1 (acute phase)**			**Model 2 (healthcare costs during follow up)**			**Model 3 (total societal costs)**		
	**Coefficient**	**T**	**P>t**	**Coefficient**	**t**	**P>t**	**coefficient**	**T**	**P>t**
Demographic									
Age	0.0003	0.12	0.904	−0.008	−1.42	0.158	−0.001	−0.45	0.653
Gender (female=1)	0.028	0.51	0.611	−0.394	−2.65	0.008	−0.094	−1.34	0.180
Living with family (vs alone/institution)	−0.010	−0.14	0.885	−0.264	−1.41	0.159	0.026	0.29	0.770
North (vs. south and centre)	0.100	1.61	0.108	−0.013	−0.07	0.942	−0.161	−1.98	0.048
Centre (vs south and north)	0.077	1.04	0.300	0.231	1.15	0.253	−0.03	−0.29	0.771
Clinical									
Diabetes mellitus	0.038	0.63	0.529	0.341	2.03	0.044	0.06	0.75	0.453
Previous stroke	0.096	1.12	0.264	−0.104	−0.44	0.663	0.128	1.15	0.253
Smoker	0.083	−1.29	0.199	0.146	0.83	0.409	−0.066	−0.79	0.432
Atrial fibrillation	0.024	0.35	0.727	0.088	0.46	0.644	0.109	1.21	0.227
Haemorragic stroke	0.010	0.13	0.896	0.486	2.41	0.016	0.160	1.69	0.092
Organizational									
Stroke Unit	0.172	2.31	0.021	0.061	0.29	0.769	0.019	0.20	0.842
Neurology ward	0.307	4.76	0.000	−0.351	−1.99	0.048	0.000	0.00	0.997
Functional outcomes*									
NIHSS (at admission)	0.014	2.57	0.011	0.027	1.82	0.069	0.020	2.93	0.004
Barthel Index categories**									
Mild dependence (75–95)	0.063	−0.78	0.434	0.202	0.98	0.329	0.220	2.25	0.025
Moderate dependence (46–74)	0.090	1.19	0.236	0.312	1.07	0.284	0.652	4.76	0.000
Severe dependence (0–45)	0.218	2.77	0.006	0.885	2.47	0.014	0.749	4.43	0.000
Modified Rankin Scale ***									
MRS 3 (moderate disability)				0.744	3.24	0.001	0.315	2.91	0.004
MRS 3–5 ( severe disability)				1.210	3.76	0.000	0.596	3.93	0.000
Constant	7.988	42.51	0.000	7.372	14.63	0.000	8.924	37.6	0.000
Number of obs	411			411			411		
R-squared	0.1617			0.3726			0.5307		
Adj R-squared	0.1227			0.3437			0.5091		
F	F( 16, 394) = 5.37		4.75				F(18,392)=24.63		
Prob > F	<0.0001			<0.0001			<0.0001		

* Scales at the beginning of the reference period.

**BI >95 (Independence) is a reference category.

*** MRS (0–2 no or slight disability) reference category.

Demographic, organizational and clinical variable do not predict social costs over the one year follow-up. All three variables used to measure functional outcome and dependency are instead associated with social costs: each point of NIHSS at admission increases social costs by 2%; according to the BI, patients with severe dependence cost 75% more than those without any dependence; moderate and severe disability patients have higher costs compared to mild disability patients by 32% and 60%, respectively.

## Discussion

To provide a national view of healthcare and societal costs of stroke survivors, we enrolled a large sample of hospitals across Italian regions. The female/male ratio, the fraction of ischemic stroke and the percentage of patients over 65 in this sample are reflective of the Italian population. To our knowledge, this is the largest cost of illness study in Italy and one of the largest conducted in Europe in the last decade [12]. In addition, this study provides original evidence about informal care over time and allows for estimates of stroke-specific costs.

Despite careful design and implementation our study presents a few limitations. Firstly, our sample was constructed to reflect major characteristics on admittance of stroke patients into hospitals and results confirm this sample representation; however centres were intentionally selected according to quotas and therefore this is not a population-based study. Secondly, although we carefully designed the collection data forms to include data attributable to stroke only, we cannot exclude that some costs associated to comobordities, rather than stroke, were computed. Thirdly, a significant number of patients lost at follow-up may present a bias as we cannot guarantee that observed and unobserved patients had the same costs (although none of the characteristics recorded was significantly different between the two groups).

In our study a stroke survivor costs €19,953 to society in the first year after the event. It is difficult to directly compare these estimates to other cost analyses available in the literature due to a variety of methods used, definition of cost components included and patient population considered. However, despite these differences, our results are similar to those of other European studies with a comparable methodology [12]. Two studies adopting a societal perspective, with a bottom up approach and one year follow-up produced estimates of € 20,239 and € 25,493 per patient in Germany and Sweden, respectively [19,20]. In a large population-based study using a German stroke registry overall direct healthcare costs for first-year survivors with primary ischemic stroke was estimated to be €18,517 [7]. The analysis was comparable in the methods for data collection but included a slightly different sample population: all hospitalized and non-hospitalized patients in the study region. Similarly, our cost estimate is also relatively close to the mean value calculated from 71 stroke studies (US $ 19,027 in 2006) [10].

Direct comparison of overall cost estimates across studies conducted in different countries is inevitably influenced by the country specific unit costs used for the evaluation of resources. Thus, a more meaningful and informative comparison can be done with physical units of resources used and relative weight of different cost categories. In this respect, our study produced very similar estimates to a study on societal costs of stroke in Germany [15]. In this study the mean length of stay of index hospitalisation was 14 days (compared to 13.5 days in our sample), and average costs of €4,650 accounted for 49% of direct costs (vs 47% in our study). Length of stay during index hospitalisation obtained in our study (13.5 ± 11.9 days) is also very similar with recently published estimates (12.8 ± 11.8 days) from the European Registry of Stroke Project (EROS) in one hospital in Florence [21].

On the basis of data of 812 stroke patients from a population based registry in Germany, rehabilitation services accounted for 37% of the total healthcare costs in the first year post event [7]. According to our estimates, the total rehabilitation costs represented 35% of total healthcare costs overall, with major concentration of costs in the first three months following the event. More specifically, in the first quarter after the acute hospitalisation about 70% of patients got rehabilitative services and this cost component absorbed about 81% of the costs of the quarter. This suggests that rehabilitation services are widely offered in Italy and that effectiveness and efficiency concerns about the provision of these services should be of paramount importance.

It is widely recognized that informal care plays a substantial role in the total care provided to stroke patients, although empirical estimates on the informal care costs associated with stroke are currently limited in European countries [12]. An international comparison of cost studies shows that informal care costs are omitted in the majority of cost of illness studies, especially when it concerns informal care time [11]. In a more recent critical review on cost of stroke studies that used patient level data, authors showed that of 120 cost studies analysed only 8 (7%) included informal care costs. Our study fulfils this gap by providing detailed estimates of informal care time and costs associated with stroke. Our estimates are in line with the results of the few similar European studies that showed these cost components as a major component on the total cost of stroke. More specifically, in a recent study that quantified the annual cost of illness of stroke in the UK, informal care accounted for 27% of total societal costs [9]. Our findings corroborate the results obtained in this study and provide important original evidence about a country which presents strong family ties and thus may have a specific attitude towards the use of informal care.

In addition to confirming mean estimates available in the literature, our study provides further information on different patterns of cost components over time. Healthcare costs change significantly over one year: while in the first quarter after the event monthly costs for healthcare amounts to €1,377 per patient, in the last two quarters the average cost per patient per month is only € 161. Conversely, non-healthcare costs tend to be more stable as they do not decrease over time. On average, a stroke patient costs €617 for informal and paid care and these two categories of costs account for about 78% of total societal costs in the second half of the observation period. Paid care and informal care costs are more persistent and may require adequate monitoring to assure that the patient benefit for appropriate social care. These results not only contribute to the existing literature on cost of stroke but also provide valuable insight on different types of data for health planning. When predicting healthcare costs, the number of events is the driver of the economic burden of disease and this implies that incident cases should be used. Contrary, for non healthcare costs the burden of the disease is driven by prevalence as these costs persist over time. While planning of hospital and rehabilitation facilities should forecast the number of incident cases, appropriate policies to assure patients receive appropriate social care should consider prevalent cases.

Our results also show that BI and mRS are good predictors of societal costs associated to stroke, specifically non healthcare costs. These findings are confirmed by previous studies and have important policy implications [16]. Social care planning could easily use these scales/indexes for predicting the amount of support that patients should receive and to provide adequate financial and in kind support.

Our estimates for the acute phase and the following six months are substantially higher than those obtained by Gerzeli and colleagues who found € 6,111 for healthcare and € 11,607 for total societal costs [5]. The two studies were designed similarly, demographic characteristics of patients are similar and there is no clear evidence that patients in our sample were more severe. It is thus reasonable to assume that differences in costs between the two studies reflect differences in intensity of care and their associated costs. Our patients cost more because of higher inpatient costs for the acute phase and greater use of rehabilitation services. It appears that in Italy stroke patients have had more access to rehabilitation services despite the fact that between 30% and 40% of our sample did not use any rehabilitation service. Further studies should be conducted to monitor the access to rehabilitation services and to investigate the reasons why a significant percentage of survivors still do not use these services.

As in the previous Italian study, we found that patients admitted to general medicine wards are less costly during the acute phase and patients admitted to neurology wards are less costly in the post acute phase. These results need to be taken cautiously as the study was not designed to test differences between admission settings. Despite controlling for various clinical and functional outcome variables we cannot exclude selection biases. Nevertheless, it is plausible to assume that the lower acute phase costs of patients admitted to medicine wards are due to less intense use of resources in this setting. To explain the lower post-acute phase healthcare costs of patients admitted to neurological wards it is more challenging. Both the hypotheses of better outcomes attributable to this setting and selection biases are plausible. More ad hoc investigation is warranted to test these hypotheses.

Our data illustrate how the Italian healthcare system supports stroke patients and are use to correlate costs to major clinical conditions, disability and severity of the disease. Our regression models confirm that clinical and functional outcome variables are good predictors of costs. Given the importance of data on routine care and available costs for cost-effectiveness analysis we urge the use of this evidence in modelling interventions to prevent or treat stroke patients.

## Conclusion

Overall, this study confirms that stroke is a major economic burden in terms of both healthcare costs and societal resources. While ageing may increase the number of incident cases and thus the need of hospital and rehabilitation infrastructures, an additional major concern regards informal care which persists in the long term and may be at risk of being underprovided given the present demographic, economic and social trends in Italy and other European countries.

## Competing interests

The authors declare that they have no competing interests.

## Authors’ contribution

All authors have made substantial contributions to conception and design of the study protocol. AS and GA were in charge of the acquisition of data, GF and AT conducted the analysis while GF, AT and VT contributed to the interpretation of data. GF and AT have drafted the manuscript and revised it critically. All authors have given final approval of the version to be published. All authors read and approved the final manuscript.

## Pre-publication history

The pre-publication history for this paper can be accessed here:

http://www.biomedcentral.com/1471-2377/12/137/prepub
